# The Risk, Need, and Responsivity Relevance of Working Alliance in a Sexual Offense Treatment Program: Its Intersection With Psychopathy, Diversity, and Treatment Change

**DOI:** 10.1177/10790632231172161

**Published:** 2023-04-24

**Authors:** Mark E. Olver, Keira C. Stockdale, Emily K. Riemer

**Affiliations:** 1 7235 University of Saskatchewan, Saskatoon, SK, Canada; 2 Saskatoon Police Service, 7235 University of Saskatchewan, Saskatoon, SK, Canada

**Keywords:** working alliance, psychopathy, sexual offense treatment, change, recidivism

## Abstract

The current study examined the self-reported working alliance of men attending a high intensity sexual offense treatment program and its associations with psychopathy, sexual violence risk, treatment change, and recidivism, in a Canadian sample of 317 incarcerated men followed up an average of approximately 10 years post release. Working Alliance Inventory (WAI; Horvath & Greenberg, 1989) self-reported total, Task, Bond, and Goal scores were positively correlated with treatment related changes in risk, and inversely associated with Psychopathy Checklist-Revised (PCL-R; Hare, 1991; Wang & Hare, 2003) scores. The Affective facet of the PCL-R, representing the callous-unemotional features of the syndrome, uniquely predicted lower Bond and Goal scores controlling for the other facets. Cox regression survival analyses demonstrated that sexual violence risk predicted increased sexual recidivism while change predicted decreased sexual recidivism controlling for PCL-R total score; however, WAI scores (particularly the Goal component) were also unexpectedly associated with increased sexual recidivism. For violent recidivism, psychopathy, risk, and change incremented the prediction of general violence, while the WAI was not significantly associated with this outcome. A set of parallel analyses, stratified by Indigenous ethnocultural heritage, demonstrated some continuity, but also potential areas of difference, in substantive findings. Risk, need, responsivity implications of the working alliance for the treatment of high psychopathy correctional clientele, and how this may intersect with Indigenous heritage, are discussed.

The working alliance (also frequently referred to as the therapeutic alliance) is a common factor shared across treatment modalities, clinical presentations, and client groups. Defined by Flückiger et al. (2018) as “a mutual collaboration and partnership between therapist and client” (p. 316), the working alliance has been identified through meta-analytic reviews as accounting for a larger portion of variability in psychotherapy outcomes than specific factors (e.g., modality, technique) (Messer & Wampold, 2002). Flückiger et al. (2018) examined 295 psychotherapy studies over a 40-year catchment period and found a small to moderate effect size (*r* = .28) between the alliance and face-to-face psychotherapy outcome that held up across different outcomes, alliance measures, assessor perspectives, patient characteristics, and treatment approaches. Arguably, the importance of the working alliance extends to correctional and forensic settings, although few studies (*k* = 3) in Flückiger et al. (2018) featured such programs.

We use the term “working alliance” to refer more broadly to all human service delivery with correctional clientele, extending to parole and probation supervision, group, and individual therapy. The risk-need-responsivity (or RNR) model of effective correctional treatment (Andrews et al., 1990; Andrews & Bonta, 2010) underpin effective risk management and reintegration services with diverse justice involved client groups (e.g., gender, ethnocultural background, development, clinical presentation, offense history). The risk principle states that the intensity of service delivery should be matched to the recidivism risk level of the client; the need principle stipulates that effective services prioritize risk-relevant changeable domains of personal and psychological functioning (i.e., dynamic risk factors or criminogenic needs) for intervention; and the responsivity principle asserts that effective services should be adapted to unique client features and is subdivided into general and specific prongs. General responsivity is captured by Andrews et al. (1990) who state, “Effective rehabilitative efforts involve workers who are interpersonally warm, tolerant, and flexible, yet sensitive to conventional rules and procedures” (pp. 36-37). These service provider features are recommended in the context of using cognitive behavioral methods to promote change. Specific responsivity entails adapting services to the unique characteristics of clientele (e.g., motivation, culture, mental health, cognitive/literacy skills). Attention to the working alliance with correctional clients is one means of heeding the responsivity principle. Meta-analysis has demonstrated failure to attend to responsivity considerations to be associated with increased attrition from correctional programs and subsequent recidivism (Olver et al., 2011), and RNR adherence to be associated with increased retention and decreased recidivism (Andrews & Bonta, 2010; Gannon et al., 2019; Papalia et al., 2019).

## Working Alliance with Correctional Clientele: Implications for RNR

In this manuscript, we operationalize working alliance as measured by variants of the Working Alliance Inventory (WAI; Horvath & Greenberg, 1989), which conceptualizes the alliance in terms of task (mutually agreed upon therapeutic activities between client and therapist), bond (emotional connection between client and therapist), and goal (mutually agreed upon outcomes or objectives by the client and therapist). This has important RNR implications. First, attention to the working alliance is attending in part to general and specific responsivity – it mitigates risk for correctional treatment dropout and maximizes the potential for client retention and gain (DeSorcy et al., 2016). In this way, the working alliance also has direct implications for the risk and need principles; individuals who are higher risk and who have greater criminogenic need may be apt to struggle with alliance formation, however, it is they who need this alliance and who stand to benefit from services the most, where we turn to next.

### Implications for Risk and Need

Research has provided some support for the notion that the working alliance has risk and need relevance; that is, individuals with positive alliances tend to do better in correctional treatment, although this is challenging with riskier clientele. Hartman and Collins (2014), for instance, found scores on the short form of the WAI (the WAI-Short Revised, or WAI-SR) to be positively associated with probation success with men under community supervision. Further, in a US SOTP sample, Blasko and Jeglic (2016) found WAI Bond scores (i.e., emotional connection between client and therapist) to be negatively correlated with a static measure of sexual violence risk, but unrelated to a measure of general recidivism risk. Finally, in a New Zealand sample of high risk men attending high intensity prison-based VOTP, Polaschek and Ross (2010) found dimensional ratings of a high risk personality disorder construct (psychopathy) to be inversely associated with observer alliance ratings on the WAI-Short Form; moreover, positive changes in therapist alliance ratings were positively correlated with treatment-related reductions in violence risk.

#### Implications for Responsivity

The responsivity relevance of the working alliance may be more evident; that is, how the alliance relates to client therapeutic buy-in, retention, and gain, particularly how this may be influenced other responsivity variables such as client perceptions of therapist characteristics, personality pathology (e.g., psychopathy), and ethnocultural diversity.

##### Therapist Characteristics

Studies in the UK examining group-based sexual offense treatment programs (SOTPs) have found positive therapist characteristics (e.g., empathy, warmth, supportiveness) to be associated with group cohesion and task orientation (Beech & Fordham, 1997) and positive client self-reported change on psychometric measures of victim blame, denial of responsibility, and minimization (Marshall et al., 2003). In a small Australian SOTP sample (*N* = 75) Watson et al. (2017) found WAI-short form scores to be positively correlated with ratings of therapist warmth and inversely with therapist interpersonal dominance. In a large Canadian sample (N = 423), DeSorcy et al. (2016) found scores on the Working Alliance Inventory (WAI; Horvath & Greenberg, 1989) were associated with successful completion of a high intensity SOTP (although unrelated to recidivism).

##### Psychopathy

The RNR implications of the working alliance also extend to psychopathy, a serious personality disorder characterized by a constellation of problematic interpersonal (e.g., deceitful, manipulative), affective (e.g., callous, unemotional), and behavioral (e.g., impulsivity, irresponsibility, poor behavioral controls) features. Psychopathy also has RNR relevance given that it predicts institutional and community recidivism (Guy et al., 2005; Leistico et al., 2008), psychopathic persons have more criminogenic needs (Simourd & Hoge, 2000), and psychopathy is associated with treatment interfering behaviors that require skilled adaptation and staff responsiveness to promote treatment retention and gain (Olver et al., 2011).

Wong’s (2015) two-component (2-C) model represents an application of RNR principles to the treatment of psychopathy (see also Hare, 2003; Wong et al., 2012), in which Factor 1 (component 1, or C-1, the interpersonal component) is a responsivity issue to be managed in treatment, while Factor 2 (component 2, C-2, the criminogenic component) represents the risk and need domains to be targeted directly in treatment. Psychopathy is characterized by interpersonal and affective features that are not conducive to forming warm, reciprocal relationships, and behavioral features that do not bode well for conscientious engaged treatment behavior. As responsivity issues, the interpersonal and affective features of Factor 1 can manifest in treatment interfering behaviors such as “staff splitting,” antagonistic, hostile and/or abrasive behavior toward staff and co-patients, manipulation and deceitfulness, failure to accept responsibility for one’s own behavior, and a lack of emotional connectedness toward helpers and in therapeutic activities. These behaviors could be construed as interfering in a relational sense, having a detrimental impact on the alliance (e.g., therapeutic ruptures). By contrast, Factor 2 represents a set of risk and need issues, given that Factor 2 (and its Lifestyle and Antisocial facets) is a stronger predictor of recidivism than Factor 1 (Yang et al., 2010) and it has high convergence with measures of risk and need (e.g., Olver et al., 2007; Simourd & Hoge, 2000). The implications per the 2-C model are to manage the treatment interfering behavior associated with Factor 1 per the responsivity principle, and to target criminogenic needs (associated with Factor 2) for risk reduction treatment per the risk and need principles. Evidence for the 2-C model come from findings showing that treatment-related changes in criminogenic needs to be associated with deceased recidivism when controlling for PCL-R scores and baseline risk, per C-2 (Olver et al., 2011; Sewall & Olver, 2019). Evidence supporting C-1 of the model comes from findings that Factor 1, in particular, the Affective facet (i.e., the callous unemotional features of psychopathy), to be associated with increased dropout (Olver et al., 2011), decreased treatment progress (Olver et al., 2011; Sewall & Olver, 2019), and weaker working alliances; this lattermost line of evidence is to where we now turn.

For instance, a New Zealand based VOTP study (Daly, 2017) of 277 men found the Interpersonal, Affective, and Lifestyle features of psychopathy to be significantly inversely associated with WAI-S Task, Bond, Goal, and total scores; Factor 1 also had stronger associations with weaker working alliance than Factor 2 per the 2-C model, given the negativistic personality features Factor 1 represents. In an SOTP sample of 89 men, Walton et al. (2018) found PCL-R total scores were negatively associated with client Bond ratings; importantly, however, the study did not report associations between PCL-R factor or facet scores with WAI ratings. Finally, in an SOTP sample of 111 men, DeSorcy et al. (2020) found PCL-R Affective facet scores were negatively associated with WAI Bond, Goal, and Total scores controlling for all other PCL-R facets, while Lifestyle facet scores were associated with lower Task, Goal, and Total scores; these findings were upheld when controlling for scores on all other facets, an important consideration, given that the facets are intercorrelated (Hare, 2003). DeSorcy et al. (2020) suggested that high Lifestyle facet scores could translate into poor therapeutic work ethic (per C-2), accounting for the negative association with Task, while the callous-unemotional features of the Affective facet would be antithetical to the formation of an emotional bond with the therapist (per C-1).

##### Ethnocultural Diversity

Finally, the working alliance intersects with ethnocultural diversity in meaningful ways. Indigenous persons, for instance, have been long overrepresented in correctional settings in Canada (Public Safety Canada Portfolio Corrections Statistics Committee, 2018), the region for the present study, as well as the US (United States Sentencing Commission, 2021), Australia (Australian Bureau of Statistics, 2021), and New Zealand (New Zealand Department of Corrections, 2021). Against a backdrop of colonization, cultural erosion, and socioeconomic disadvantage (Truth and Reconciliation Commission of Canada, 2015), these circumstances pose anticipated challenges to the formation of a strong working alliance, particularly with White nonminority service providers. Little research, however, has examined the working alliance with Indigenous correctional clientele. DeSorcy et al. (2016) found Indigenous men attending a high intensity sexual violence reduction program had lower WAI Bond scores than non-Indigenous men. This may be anticipated, given the systemic, historical, and experiential considerations of Indigenous persons involved in the justice system, which can translate into mistrust and skepticism toward service providers, particularly when the services providers are not of Indigenous heritage. Interestingly and perhaps unexpectedly, however, in a follow-up study with a subsample rated on the PCL-R, DeSorcy et al. (2020) found that high psychopathy scorers generally had lower WAI scores than low psychopathy scorers, but this was most evident for non-Indigenous men.

#### Current Study and Rationale

There are remarkably few empirical studies examining the working alliance with correctional clientele and fewer still have examined associations with: (1) clinically relevant constructs such as psychopathy and risk, or (2) with critical outcome measures such as treatment change and criminal recidivism, or (3) examined clinical and outcome ramifications of the working alliance with respect to ethnocultural diversity. As such, the current study examined the self-reported working alliance of men attending a high intensity sexual offense treatment program (SOTP), and its associations with psychopathy, sexual violence risk, treatment change, and recidivism; the results of which have implications for Wong’s (2015) 2-C model. Given the overrepresentation of Indigenous persons in Canadian corrections, these associations were examined within the context of Indigenous heritage.

Relatedly, the use of mainstream forensic measures to assess risk and need with persons of Indigenous ancestry has been a source of legal challenges and debate (Canada v. Ewert, 2015; Ewert v. Canada, 2016; Ewert v. Canada, 2018), and how such measures, including the working alliance, intersect with Indigenous ancestry among men attending SOTP can have significant treatment planning and delivery implications. Although research has marshalled support for the structural and predictive properties of certain tools such as the PCL-R (Olver et al., 2018a), Violence Risk Scale-Sexual Offense version (VRS-SO; Olver et al., 2018b), and Static-99R (Lee et al., 2020) with Indigenous persons, this has not quelled the debate, and the need to demonstrate the utility of these measures in applied clinical and correctional contexts with Indigenous persons remains. The following hypotheses were proposed:

1. WAI scores would be meaningfully associated with responsivity indicators, specifically, higher levels of cognitive ability and inversely associated with diagnoses of personality disorder in general and antisocial personality disorder (ASPD) in particular.
2. WAI scores would be positively correlated with sexual offense treatment change, measured pre-posttreatment, by the VRS-SO, a dynamic risk assessment and treatment planning tool.
3. PCL-R scores would be inversely associated with WAI scores, particularly the PCL-R Affective facet with WAI Bond scores, through bivariate and multivariate analyses.
4. Psychopathy and sexual violence risk would predict increased sexual and violent recidivism, while WAI score and treatment-related change would be associated with decreased recidivism.
5. Power permitting, and in light of prior research (DeSorcy et al., 2020; Olver et al., 2018a, 2018b) findings generated from hypotheses 2-4 would have consistency in the magnitude of effect and direction of findings in targeted parallel analyses stratified by Indigenous and non-Indigenous ethnocultural subgroup. Specifically, we proposed:
  a. VRS-SO change scores would be positively associated with WAI scores within stratified Indigenous and non-Indigenous subgroups.
  b. PCL-R scores, particularly the Affective, facet would be significantly inversely associated with WAI scores within stratified Indigenous and non-Indigenous subgroups.
  c. PCL-R and VRS-SO scores would be associated with increased sexual and violent recidivism, while WAI and VRS-SO change scores would be associated with decreased sexual and violent recidivism, within stratified Indigenous and non-Indigenous subgroups.

## Method

The present research received ethical approval from the University Behavioural Research Ethics Board (Beh REB #15-366) and operational approval from the Correctional Service of Canada (CSC). In accordance with the 21-word solution from Simmons et al. (2012), “We report how we determined our sample size, all data exclusions (if any), all manipulations, and all measures in the study” (par. 6).

### Participants and Sample Description

The sample featured 317 men serving federal sentences (i.e., 2 years or greater) primarily for contact sexual offenses (99.4%), referred to a high intensity sexual offense treatment program (SOTP) at a maximum security correctional mental health facility operated by CSC; the remaining 0.6% of cases were serving federal sentences for nonsexual offenses but had unadjudicated histories of sexual offending. The men were admitted to the program between 1994 and 2003 (median year 1999) and were 37.4 years of age at admission (*SD* = 10.4, *n* = 315), and 38.9 years of age (*SD* = 10.3, *n* = 275) at community release. The combined sample consisted of three sets of treatment cohorts: 1994-1997, 1998-2000, and 2001-2003. The men were serving a mean sentence length of 5.9 years (*SD* = 3.8, *n* = 290) for their current sexual offense(s), most of whom (71.3%, 219/307) had a formal criminal history of sexual offending. Approximately two thirds (65.9%, *n* = 209) of the sample had at least one adult victim, 30.9% (*n* = 98) had exclusively child victims, and this was unknown for 3.1% (*n* = 10). Nearly half (44.5%, *n* = 141) of the sample was of Indigenous descent, while the remainder was White or other ethnic descent (54.6%, *n* = 173), or unknown (0.9%, *n* = 3). Over half the men were currently/previously married or equivalent (56.8%, 180/308) and 40.4% (128/308) were single/never married.

In terms of level of sexual violence risk and need, most participants (41.6%, 114/274) were classified as Well Above Average (Level IVb) or Above Average (Level IVa; 34.7%, 95/274) risk on the Static-99R, with the remainder classified as Average (Level III; 19.7%, 17/274) or Below Average (Level II; 4%, 11/274) risk. On the VRS-SO (see instrument description below), 14.9% (42/282) of cases were classified as Well Above Average risk (Level IVb) and nearly half (45.7%, 129/282) were classified as Above Average risk (Level IVa), with about one third (34.4%, 97/282) at Average (Level III), and the remainder at Below Average (Level II; 4.3%, 12/282) or Well Below Average (Level I; 0.7%, 2/282) risk.

### Treatment Program Description and Statistics

The SOTP, which was known as the Clearwater Program, was an 8–9-month high intensity cognitive behavioral treatment program for men convicted for sexual offenses. The program’s mission was to reduce risk for future sexual offending and promote successful community reintegration, characterized by sexually and relationally healthier lives without sexual violence. Program referrals were typically appraised as high risk for future sexual violence and/or had personal or psychological concerns that warranted a high intensity program. The SOTP operated on a closed unit with group and individual services prioritizing risk-relevant domains that included problems with intimacy and relationships, emotional regulation, attitudes supportive of sexual offending, sexual deviance/self-regulation, as well as healthy sexuality, among other clinical foci. The SOTP content advanced with developments in knowledge and practice concerning effective assessment, intervention, prevention, and risk management of sexual violence (Olver et al., 2011). The program was delivered by a multidisciplinary team of service providers including psychiatric nurses who provided the bulk of individual and group therapy, in addition to psychologists, psychiatrists, occupational therapists, social workers, parole and correctional officers. Moreover, owing to the overrepresentation of Indigenous persons in Canadian corrections, Indigenous Elders featured prominently in treatment, consultation, and provision of cultural services (e.g., smudging, sweat lodges).

The average length of stay in the program (*n* = 312) was 7.8 months (*SD* = 5.1) with 85.3% (267/313) of men successfully completing the program (i.e., identified as completing substantive program requirements and receiving a successful completion). As would be expected, treatment completers had significantly longer duration in treatment (*M* = 8.1, *SD* = 5.4) than noncompleters (*M* = 5.8, *SD* = 2.1), t (308) = 2.77, *p* = .003. Program participants (*n* = 270) were on average 31.7 months (*SD* = 32.9) into their sentence prior to program admission, and remained in custody an average of 16.6 months (*SD* = 19.9) prior to community release. Previous program evaluations of the SOTP have demonstrated an approximate 30–50% reductions in sexual recidivism for treatment attendees relative to matched untreated controls (Olver et al., 2009).

### Measures

#### Working Alliance Inventory

We employed the self-report version of the Working Alliance Inventory (WAI; Horvath & Greenberg, 1989), a 36-item measure of the strength and quality of the therapeutic relationship between client and therapist. Each item is scored on a seven-point Likert scale ranging from 1 (Never) to 7 (Always). Possible scores range from 36 to 252 with higher scores reflecting stronger alliances. Items can be summed to generate a total score and can also be arranged into three 12-item subscales measuring Task (i.e., to what extent sessions are characterized by relevant tasks or interventions), Bond (i.e., to what extent a warm, empathic emotional connection exists between client and therapist), and Goal (i.e., to what extent the client and therapist share mutual objectives and anticipated outcomes with treatment). Short forms (WAI-S; Tracey & Kokotovic, 1989), offender adapted short forms (WAI-SR; Tatman & Love, 2010) and observer rated (WAI-O; Tracey & Kokotovic, 1989) versions of the WAI also exist. The self-report version of the WAI was instituted in the SOTP in the early 1990s given its convenience of administration and utility to assess the quality of alliance from the patient’s point of view. In the current sample of 317 treated men, the subscales were highly intercorrelated (all *p* < .001): Task X Bond *r* = .70; Bond X Goal *r* = .74; and Task X Goal *r* = .86. We examined the three subscales in our analyses given that the domains conceptually are intended to measure different nuances of the working alliance.

#### Violence Risk Scale-Sexual Offense version

The Violence Risk Scale-Sexual Offense version (VRS-SO; Wong et al., 2003, 2012) is a clinician rated sexual violence risk assessment and treatment planning tool. It is designed to assess risk for sexual violence, identify targets for risk reduction intervention, evaluate change on identified targets, and to inform post intervention risk management. The VRS-SO is unique from other dynamic sexual violence risk tools given that it has a mechanism to explicitly evaluate change and corresponding risk reduction from treatment or other change agents. The VRS-SO is comprised of seven static (i.e., historical, generally unchanging) and 17 dynamic (i.e., potentially changeable interpersonal, social, and psychological characteristics) items linked to sexual violence risk. Each item is scored on a 4-point (0, 1, 2, 3) ordinal scale; higher scores on the static items reflect density and versatility of offense history, while higher dynamic item scores represent problem areas linked to sexual offending. Items rated two or three are considered criminogenic and to be prioritized for treatment; items with 0 or one rating are low risk items, or possibly strengths. Factor analyses have demonstrated the dynamic items can be arranged into three factors: Sexual Deviance (e.g., deviant sexual preference, sexual compulsivity, sexually deviant lifestyle), Criminality (e.g., interpersonal aggression, substance abuse, impulsivity), and Treatment Responsivity (e.g., cognitive distortions, insight) (Olver et al., 2007).

Change is evaluated through a modified application of the transtheoretical model of change or stages of change (Prochaska et al., 1992), representing cognitive, experiential, and behavioral changes as a client attempts to ameliorate problem areas. The five stages are operationalized for each dynamic item: precontemplation (i.e., denial of problem area, lack of insight), contemplation (i.e., acknowledgement of problem area), preparation (i.e., emergence of cognitive and behavioral skills and strategies to manage problem area), action (i.e., sustained use of skills and strategies over an extended period), and maintenance (i.e., generalization and transfer of new skills across different contexts and tested in high risk situations). Progression from one stage to the next in the direction of risk reduction is associated with a 0.5-point deduction, two stages, 1.0-points, and so on; the exception is movement from precontemplation to contemplation, which is not credited with a score deduction given that there is no observable behavioral change. Deterioration between stages is associated with a corresponding increase in score. The change ratings are summed across all dynamic items to generate a change score; this is deducted from the pretreatment score to generate a posttreatment score.

The VRS-SO was instituted for routine use within the SOTP in the early 2000s with clinician ratings comprising about one quarter of the sample (*n* = 76) and the remainder (*n* = 206) rated from file. The VRS-SO was independently double coded from file in two archival studies from which the present sample was culled and demonstrated acceptable interrater reliability in each: VRS-SO dynamic total score, single measure, consistency agreement, ICC_C1_ = .74 (pretreatment) and .79 (posttreatment), *n* = 35 (Olver et al., 2007); and ICC_C1_ = .86 (.73 with outlier, pretreatment) and .87 (.74 with outlier, posttreatment), *n* = 21 (Sowden & Olver, 2017).

#### Psychopathy Checklist-Revised

The Psychopathy Checklist-Revised (PCL-R) is a 20-item symptom construct rating scale designed to assess the construct of psychopathy and is considered the gold standard (Spies & Plake, 2005). Items are rated on a 3-point ordinal scale (0 = not present, 1 = partially/possibly present, 2 = present) and summed to yield a total score from 0-40 representing the extent to which the individual resembles a prototypically psychopathic individual. Research demonstrates that valid and reliable ratings of the PCL-R can be obtained from quality information from comprehensive institutional files alone or when accompanied by other collateral sources and a semi-structured interview (Hare, 2003; Wong, 1984; 1988). Although research demonstrates psychopathy to be a dimensional construct (Edens et al., 2006), cut scores ranging from 25 (file only) to 30 (file plus interview) have been used to characterize psychopathy. Research has demonstrated that file-based ratings can generate slightly conservative estimates of the PCL-R total score for high scoring individuals (Wong, 1984; 1988), and as such, file-based rating cutoffs around 25 have been employed to characterize high psychopathy for research purposes (e.g., Beggs & Grace, 2008; Sewall & Olver, 2019; Wong et al., 2012). These lines of research show that the PCL-R can be rated reliably with or without the interview, and that the two show good agreement when quality information is available (Wong, 1988).

Results from confirmatory factor analysis demonstrate that the PCL-R items can be organized into two factors and four facets (Hare & Neumann, 2008). In the two-factor model, Factor 1 refers to a set of interpersonal and affective features, while Factor 2 represents the chronic antisocial lifestyle. Factor 1 can be subdivided into two first order factors or facets termed Interpersonal (e.g., superficial, grandiose, deceitful, manipulative) and Affective (e.g., lack of remorse, callous lack of empathy). Factor 2 can similarly be organized into Lifestyle (e.g., parasitic, impulsivity, irresponsibility) and Antisocial (e.g., poor behavioral controls, release failure, criminal versatility) facets. Research supports the predictive validity of the PCL-R for community recidivism (Leistico et al., 2008), future violence (Campbell et al., 2009), and institutional misconducts (Guy et al., 2005). The PCL-R was independently double coded across three studies from which the present sample was culled, demonstrating strong interrater reliability in each: PCL-R total score, single measure absolute agreement, ICC_A1_ = .85, *n* = 21 (DeSorcy et al., 2020); ICC_A1_ = .87, *n* = 35 (Sewall & Olver, 2019); and ICC_A1_ = .84, *n* = 32 (Olver & Riemer, 2021).

#### Additional Responsivity Indicators

Meta-analytic research has demonstrated that lower levels of education, employment, intellectual ability and well as certain categories of psychiatric diagnosis (e.g., antisocial personality disorder, any personality disorder, psychosis) to be associated with increased risk for drop out from offender treatment programs (Olver et al., 2011). As such, adapting service delivery in response to these client characteristics, per the responsivity principle, may mitigate risk for noncompletion. In this study, we identified two additional sets of responsivity indicators, cognitive functioning and mental health diagnosis, given that these have the potential to influence the course of services, which in turn need to be adapted to maximize client gain and minimize attrition. Three self-report measures of cognitive functioning were employed: Raven’s Progressive Matrices, a measure of nonverbal problem solving (Raven, 1938); the Symbol Digit Modality Test (SDMT), a measure of cognitive processing speed (Smith, 1973); and the Quick Test, a test of receptive vocabulary that provides an index of verbal ability (Ammons & Ammons, 1962). The second set of responsivity indicators was binary presence or absence of a Diagnostic and Statistical Manual for Mental Disorders (DSM IV or IV-TR) diagnosis, as assigned upon program intake by psychiatry staff.

#### Recidivism

Recidivism was captured through officially reported criminal charges and convictions from Canada’s nationwide criminal record database, the Canadian Police Information Centre (CPIC), maintained by the Royal Canadian Mounted Police (RCMP). CPIC registers new criminal charges and convictions incurred throughout the country and has the advantage of being continually updated and its nationwide coverage. Recidivism was coded in a binary manner (1 yes recidivate, 0 no recidivate) in addition to the disposition date for the recidivistic event (i.e., charge or conviction date). Further, periods of pretrial custody prior to sentencing (when this information was available) were subtracted from the survival time variable to yield a more accurate estimate of time to reoffense in the community. Two of the treatment study cohorts (Olver et al., 2007; Sowden & Olver, 2017) employed the more conservative recidivism criterion of conviction; the third and most recent cohort included charges as well as convictions in the operationalization of sexual recidivism (due to unusually low base rates related to backlogs with RCMP criminal record processing at the time; see (Olver et al., 2020) in order to yield more accurate base rate estimates and greater statistical power. Sexual recidivism extended to any new sexually motivated offenses (e.g., sexual assault, invitation to sexual touching), including noncontact sexual offenses (e.g., child pornography). Offenses that were adjudicated as nonsexual offenses (e.g., nonsexual assault, murder) that could be determined to be sexual in nature (e.g., sexual homicide) were also coded as new sexual offenses. Violent recidivism was defined as any new offense against the person with the potential to cause physical or psychological harm (e.g., uttering threats, robbery, assault) including sexual offenses; thus, the two categories were not mutually exclusive, as the broader violent recidivism category encompassed both sexual recidivism and nonsexual violent recidivism in order to capture globally interpersonally harmful criminal behavior toward others.

### Procedure

The present study is part of a larger ongoing program of research examining SOTP treatment responses and outcomes as a function of PCL-R measured psychopathy. The present sample was selected by identifying individuals who had completed the WAI from three treatment study cohorts (Olver et al., 2007, 2020; Sowden & Olver, 2017), each of which were consecutive admissions to the SOTP. The sample was further narrowed by limiting those cases for inclusion that had either VRS-SO or PCL-R ratings available. The VRS-SO had been rated from previous projects, either by research assistants from file (Olver et al., 2007; Sowden & Olver, 2017) or by clinicians with interview as part of SOTP services (Olver et al., 2020). PCL-R ratings, in turn, had either been extracted from previous archival studies (*n* = 134; Sewall & Olver, 2019) or were completed anew for the purposes of the present study (*n* = 123); all PCL-Rs were completed from file review. The research assistants were trained clinical psychology graduate students or senior undergraduate psychology honors students who received training on the VRS-SO and PCL-R from the first author, a registered psychologist, who was consulted as needed in order to resolve any scoring issues or ambiguities. Different sets of raters completed the VRS-SO and PCL-R ratings, all of whom were blind to WAI scores and recidivism outcome. As reported in the respective instrument descriptions, IRR ratings were completed for the VRS-SO and PCL-R on randomly selected double coded cases in each of the separate study samples from which the data were extracted (see instrument descriptions for ICC values). Finally, recidivism data were obtained via CPIC, as noted previously, and were coded by the first author, a trained graduate student researcher, and a staff psychologist serving as Director of Research at the institution. All coders followed a common set of coding rules (i.e., operationalization and identification of sexual and violent recidivistic offenses) and consulted to address coding ambiguities; all recidivism coding activities were overseen by the first author.

In all, 317 men had completed the WAI, of whom 282 had VRS-SO ratings, 257 had PCL-R ratings, and 222 had ratings on all three measures as well as recidivism outcome. As such, there were missing data due to the fact that the data were extracted across independent treatment study samples, and not necessarily every measure had been rated in a given project that employed one of the samples for different reasons (e.g., not all cases that had ratings on one measure in an earlier study could be retrieved [e.g., due to pardons, etc.] to rate another measure in a different, later study). All analyses employed listwise deletion given these circumstances of the missing data.

The WAI and measures of cognitive ability were routinely administered to all new admissions to the SOTP within the first 3 months of the program. The time duration allows for the formation of a working alliance and the opportunity to repair ruptures and respond to client feedback to strengthen the alliance. The measures were administered by a research psychometrist to the men for each treatment cohort. The staff member supervised the administration of each measure and was available to answer questions. Upon completing the WAI specifically, the men were instructed to evaluate the quality of their relationship with their assigned primary therapist on the unit, who was most frequently a psychiatric nurse or social worker. Hardcopies of the test protocols were stored separate from the men’s clinical files (and hence not available to raters coding the VRS-SO and PCL-R), entered into a spreadsheet electronically; all entries were crosschecked by the first author.

## Planned Analyses

Given that a comparative examination between Indigenous and non-Indigenous men has been conducted on the alliance with these WAI ratings (DeSorcy et al., 2016), although not in relation to the other study measures, the primary analyses were conducted on the aggregate sample to maximize power. A set of ethnoculturally stratified analyses were also conducted, however, and presented in supplemental materials. Owing to limited power and to control familywise error from multiple tests, the supplemental ethnocultural analyses were limited to testing only the most essential components of core research questions from hypotheses 2-4 pertaining to working alliance, psychopathy, risk, and recidivism as outlined in the set of hypotheses under hypothesis 5.

First, as one examination of the responsivity properties of the working alliance, WAI total and subscale scores were correlated with a set of demographic, cognitive functioning, and diagnostic variables. Meta-analytic research (Olver et al., 2011) demonstrates several of these variables to be linked to treatment completion and recidivism, and their associations with WAI scores could have implications for the formation and maintenance of the alliance. Correlation magnitudes for continuous variables were interpreted using Cohen’s (1992) conventions in which values of .10, .30, and .50 correspond to small, medium, and large effects, respectively. Second, WAI total and subscale scores were correlated with VRS-SO risk and change scores to examine to what extent positive alliance with the primary service provider was associated with the client’s risk level (i.e., could high risk men form positive alliances?) or linked to markers of treatment progress.

Third, given the limited psychopathy and working alliance research, bivariate correlations were computed between PCL-R total and facet scores with WAI indices, and in essence examine C-1, the interpersonal component of Wong’s (2015) 2-C model for the treatment of psychopathy. Given that some facets (e.g., Affective) may have unique implications for the alliance, a series of multiple regression analyses were conducted with simultaneous entry of the four facets predicting WAI total and subscale scores. As psychometric research demonstrates that the four facets are intercorrelated (Hare, 2003), partialling out variance shared with other facets may add conceptual clarity to interpreting facet associations with outcome. Lynam et al. (2006) note that partialling may aid interpretation when the predictor variables represent narrow constructs and the resulting partialled associations are smaller in magnitude than the unpartialled ones. Hicks and Patrick (2006) also alert for the possibility of suppressor effects, which would be evident if partialled associations increased substantially in magnitude. We heeded these analytic and interpretive considerations with resultant findings.

Finally, a series of Cox regression survival analyses were conducted to examine to what extent the alliance may be predictive of criminal recidivism post release, particularly for sexual or other violent crimes, controlling for individual differences in psychopathy, risk, and treatment change. In these analyses, PCL-R total scores, VRS-SO pretreatment total and change scores, and WAI total scores were entered simultaneously and their unique associations with sexual and violent recidivism over time were examined. In these analyses, we use total scores for all measures given that they provide the most comprehensive and representative measure of their targeted construct for the purposes of these analyses. In supplemental analyses, each of the WAI subscale scores were substituted in place of the total score to examine the possibility that certain aspects of the alliance may have differential associations with outcome. The Cox regression findings have implications for C-2 of Wong’s (2015) 2-C model, that is, treatment related changes in domains of risk and need should be associated with decreases in recidivism, even when accounting for individual differences in levels of psychopathy. For these analyses, treatment cohort was entered as a stratum variable to control for possible cohort effects influencing predictor-criterion variable relations. Cox regression generates a hazard ratio (e^B^) corresponding to the percent change in the hazard of an event (e.g., recidivism) per one-unit change in the predictor; values below 1.0 indicate an inverse association between the predictor and criterion, while values above 1.0 represent a positive association. In addition to the multivariate model, we computed Harrell’s C to report overall bivariate associations between each model predictor and recidivism over time. Harrell’s C is analogous to the area under the curve (AUC) statistic, with C values representing the probability that given two randomly selected cases, the individual with the higher score will recidivate sooner. With C values of .50 representing chance level probability, we use Rice and Harris’ (2005) AUC interpretation guidelines in which values of .56, .64, and .71 correspond to small, medium, and large effects, respectively. All analyses were conducted using SPSS for Windows version 25.0, with the exception of Harrell’s C, which was computed in R version 4.0.0 using the “Survival” package (Version 3.1-11; Therneau, 2020).

## Results

### Working Alliance and Associations with Responsivity Considerations

Table 1 reports descriptive statistics for the responsivity indicators and their bivariate associations with WAI scores. As a whole, the sample was broadly average cognitive ability, with some high school education, and about half having some stability in prior employment. Major mental disorder diagnoses were less common, although about one third had a paraphilia diagnosis, half had a diagnosis of ASPD, and about two thirds each had a diagnosis of SUD or any PD. Verbal ability (Quick Test score) and employment stability had significant positive correlations (approaching moderate in magnitude) with strength of working alliance across all WAI indices. Nonverbal reasoning (Raven’s) ability was significantly associated with WAI Task, Goal, and total scores while processing speed (SDMT), with the Goal subscale only (all small in magnitude effects); no other correlations were significant. Years of education was not meaningfully associated with WAI score. With the exception of psychotic illness, binary diagnosis generally had weak, nonsignificant associations with working alliance indices.

**Table 1.** WAI Correlations with Responsivity, Sexual Violence Risk, and Psychopathy, Measures.

Measure	*n*	*M (SD)/%*	WAI Measure *(r)*			
			Task	Bond	Goal	Total
Cognitive/vocational						
Raven’s (percentile)	174	55.7 (32.4)	.19*	.12	.17*	.17*
SDMT (std deviation units)	173	−0.74 (1.2)	.14	.06	.17*	.13
Quick test (percentile)	172	36.2 (26.4)	.24**	.19*	.27***	.25***
Education (years)	233	9.6 (2.8)	.13	.05	.11	.10
Employment (binary)	202	54.5	.23***	.26***	.19**	.25***
Diagnostic						
Mood	235	18.3	−.07	−.10	−.10	−.10
Psychosis	235	4.3	−.21***	−.16*	−.17**	−.20**
Anxiety	235	3.8	.01	.01	.00	.01
Paraphilia	235	31.9	.07	.09	.02	.07
Substance use	235	63.4	.04	−.01	.06	.03
Antisocial PD	235	44.3	−.06	−.12	−.06	−.09
Any PD	235	65.5	−.04	−.09	−.04	−.07
VRS-SO						
Pretreatment						
Static total	285	11.1 (3.7)	−.12	−.08	−.04	−.09
Sexual deviance	282	8.4 (4.0)	.04	−.02	.05	.02
Criminality	282	10.4 (3.6)	−.03	−.06	.01	−.03
Treatment responsivity	282	7.1 (2.4)	−.19***	−.20***	−.22***	−.22***
Dynamic total	282	29.8 (6.8)	−.06	−.11	−.03	−.08
Pretreatment total	282	40.9 (9.1)	−.10	−.11	−.04	−.09
Posttreatment						
Sexual deviance	282	7.3 (3.5)	−.02	−.05	−.01	−.03
Criminality	282	9.1 (3.3)	−.09	−.12*	−.06	−.10
Treatment responsivity	282	5.9 (2.4)	−.27***	−.26***	−.28***	−.29***
Dynamic total	282	25.7 (6.9)	−.14*	−.17**	−.11	−.16**
Posttreatment total	282	36.8 (9.0)	−.17**	−.16**	−.11	−.15**
Change						
Sexual deviance	282	1.1 (1.1)	.17**	.09	.18**	.15**
Criminality	282	1.3 (1.1)	.14*	.11	.15*	.14*
Treatment responsivity	282	1.2 (1.0)	.15*	.13*	.10	.14*
Dynamic total	282	4.1 (2.8)	.20***	.15*	.19***	.19***
PCL-R						
Interpersonal	256	2.8 (2.0)	.01	−.08	.00	−.03
Affective	257	4.5 (2.1)	−.10	−.20**	−.16*	−.17**
Lifestyle	257	5.7 (2.5)	−.10	−.17*	−.10	−.14*
Antisocial	257	5.5 (2.7)	−.08	−.13	−.02	−.09
Factor 1	257	7.3 (3.6)	−.06	−.16**	−.10	−.12
Factor 2	257	11.1 (4.6)	−.10	−.17*	−.07	−.13*
Total	257	20.7 (7.2)	−.07	−.19**	−.07	−.12*
WAI *M (SD)*	317	—	67.5 (11.9)	61.4 (15.6)	64.6 (12.6)	193.5 (35.8)

*Note.* ***p ≤ .001, **p < .01, *p < .05. WAI: Working Alliance Inventory; VRS-SO: Violence Risk Scale-Sexual Offense version; PCL-R: Psychopathy Checklist-Revised; SDMT: Symbol digit modality test; PD: personality disorder.

### Working Alliance Associations with Sexual Violence Risk, Need, and Change

Table 1 also reports descriptive statistics and bivariate correlations between VRS-SO dynamic total and factor pre, post, and change scores with WAI total and subscale scores. Most pretreatment and posttreatment dynamic measures, indicating level of sexual violence risk, had weak and nonsignificant associations with WAI indices. An exception was the Treatment Responsivity factor, in which higher WAI scores overall and on the Task, Bond, and Goal subscales were associated with lower scores at pre and posttreatment on this factor. In addition, posttreatment dynamic scores had small significant associations with each WAI index, with the exception of the Bond subscale; this reflected an overall trend for posttreatment associations to more frequently be in the expected direction and slightly higher in magnitude than pretreatment associations.

On average, VRS-SO dynamic scores changed by approximately four points, more than half a standard deviation (*d* = .60), which equates to reducing risk on about 8/17 VRS-SO dynamic items. The 1-point of change or thereabouts as reflected in the change score on each broad factor (e.g., Sexual Deviance) reflects movement in the direction of risk reduction on about two items for each of the factors. As seen by the magnitude of the SD for all change measures, there was considerable variability in the amount of pre-post change registered in the dynamic domains of the instrument. VRS-SO change scores, in the direction of improvement (i.e., risk reduction) were positively associated with WAI scores; this was demonstrated for both Indigenous and non-Indigenous subgroups, with some notable exceptions (see Table 2). Decreases in Sexual Deviance and Criminality scores from pre to posttreatment were most frequently associated with stronger working alliances at pretreatment, overall, and on the Task and Goal subscales; meaningful associations were found for changes on Sexual Deviance for the non-Indigenous subgroup only, however. Changes on the dynamic total score had significant, small in magnitude associations, with each WAI index, while positive changes in Treatment Responsivity were only meaningfully associated with pretreatment Task scores. For Indigenous men, changes on the dynamic total were meaningfully associated with Task and Goal scores only, while associations with Bond (for all change scores) tended to be weak and non-significant. By contrast associations of VRS-SO change with Bond and total scores tended to be meaningful in significance and magnitude for non-Indigenous men only.

**Table 2.** WAI Correlations with VRS-SO Change Scores among Indigenous and Non-Indigenous Subgroups.

VRS-SO change measure	*M (SD)*	WAI Measure (*r*)			
		Task	Bond	Goal	Total
Indigenous					
Sexual deviance	0.93 (1.1)	.09	−.04	.14	.06
Criminality	1.5 (1.1)	.15	.11	.15*	.14
Treatment responsivity	1.3 (1.0)	.15	.13	.06	.08
Dynamic total	4.2 (2.8)	.17*	.04	.15*	.12
Non-indigenous					
Sexual deviance	1.2 (1.2)	.21**	.15*	.21**	.21**
Criminality	1.2 (1.2)	.16*	.14	.16*	.16*
Treatment responsivity	1.2 (1.0)	.15*	.22*	.14*	.18*
Dynamic total	4.1 (2.9)	.22**	.23*	.22**	.24***

*Note.* ***p ≤ .001, **p < .01, *p < .05 (one tailed test). Indigenous *n* = 127; Non-Indigenous *n* = 153.

### Working Alliance and Psychopathy

**Figure 1.**
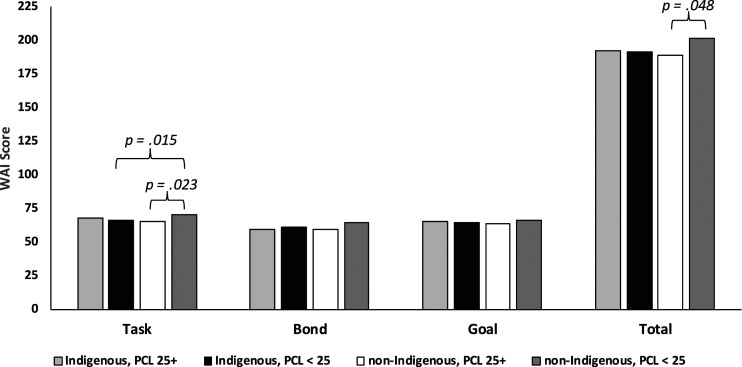
Graphic display of factorial MANOVA of WAI score by Indigenous X PCL-R group.

Table 1 reports descriptive statistics and bivariate associations between PCL-R total, factor, and facet scores with WAI total and subscale scores in the aggregate sample. On average the sample demonstrated moderate levels of psychopathy with a PCL-R total score around 20; about 1/3 of the sample (83/256) had PCL-R cut scores of 25 or higher suggesting high levels of psychopathy. PCL-R Affective facet scores had the most consistent pattern of meaningful associations with WAI score, with significant, small in magnitude, bivariate correlations with WAI total, Bond, and Goal scores. In ethnoculturally stratified analyses, the mainstay between the subgroups was a significant inverse association between Affective facet and Bond scores (Table 3). PCL-R Factor 1, Factor 2, and total scores each had small significant inverse associations with Bond scores in the sample as a whole; no associations with the remaining WAI subscale or total scores were significant. As seen in Figure 1, comparing high and low PCL-R groups as a function of Indigenous and non-Indigenous group membership through a factorial MANOVA, demonstrated small to below threshold effects observed in the direction of low PCL-R groups scoring higher on the WAI; the most notable differences were higher WAI total and Task scores for low PCL-R scoring men within the non-Indigenous subgroup. Of note, the high PCL-R group did not have significantly higher rates of treatment noncompletion (15.7%, 13/83) than the low PCL-R group (14.5%, 25/173), (χ2 [*N* = 256, 1] = 0.07, *p* = .799, φ = .02); although encouraging, this does not take into account attrition occurring prior to the administration of the WAI or refusal to complete the measure.

**Table 3.** WAI Correlations with PCL-R Facet and Total Scores among Indigenous and Non-Indigenous Subgroups.

PCL-R Measure	*M (SD)*	WAI Measure *(r)*			
		Task	Bond	Goal	Total
Indigenous					
Interpersonal	2.4 (1.9)	.10	−.03	.08	.05
Affective	4.4 (2.2)	.01	−.17*	−.06	−.09
Lifestyle	6.1 (2.4)	.00	−.10	−.12	−.08
Antisocial	6.0 (2.4)	−.07	−.14	−.08	−.11
Total	21.3 (7.1)	−.06	−.12	−.02	−.04
Non-Indigenous					
Interpersonal	3.1 (2.0)	−.09	−.16*	−.08	−.12
Affective	4.5 (2.1)	−.20**	−.23**	−.25**	−.25**
Lifestyle	5.4 (2.6)	−.15*	−.20**	−.07	−.16*
Antisocial	5.0 (2.9)	−.07	−.10	.01	−.06
Total	20.2 (7.3)	−.15*	−.23**	−.11	−.18*

*Note.* **p < .01, *p < .05 (one tailed test). Indigenous *n* = 115; Non-Indigenous *n* = 141.

Table 4 reports the results of regression examining the unique associations between PCL-R facets and WAI score. Only the Affective facet meaningfully incremented predictions of working alliance (Bond, Goal, and total score) controlling for scores on the remaining three facets. Among ethnocultural subgroups, only the Affective facet was meaningfully uniquely associated with Bond score, although the multipredictor models were underpowered owing to the decrease in cell size (Table 5). In the aggregate sample, Lifestyle facet scores had significant small in magnitude correlations with Bond and WAI total score but did not incrementally predict any alliance scores. Interpersonal and Antisocial facet scores had weak and nonsignificant associations with WAI scores at all steps of analysis.

**Table 4.** Multiple Regression: Multipredictor PCL-R Facet Associations with WAI Score.

PCL-R Facet Score Regression model	*Regression and WAI DV*			
	*B*	*SE*	*β*	*p*
Model 1	WAI task			
Interpersonal	.557	.428	.095	.194
Affective	−.664	.428	−.120	.122
Lifestyle	−.275	.365	−.060	.453
Antisocial	−.169	.316	−.040	.594
Constant	71.896	2.125		
Overall regression model	*R* = .15 *R*^*2*^ = .02. *F* (4, 251) = 1.47, *p* = .210			
Model 2	WAI bond			
Interpersonal	.257	.512	.036	.617
Affective	−1.151	.513	−.171	**.026**
Lifestyle	−.424	.437	−.076	.333
Antisocial	−.277	.378	−.053	.465
Constant	70.277	2.544		
Overall regression model	*R* = .22, *R*^*2*^ = .05. *F* (4, 251) = 3.32, *p* = .011			
Model 3	WAI goal			
Interpersonal	.670	.442	.110	.131
Affective	−1.142	.443	−.198	**.010**
Lifestyle	−.315	.378	−.065	.405
Antisocial	.150	.327	.034	.646
Constant	69.289	2.198		
Overall regression model	*R* = .19, *R*^*2*^ = .04. *F* (4, 251) = 2.45, *p* = .047			
Model 4	WAI total			
Interpersonal	1.483	6.270	.085	.241
Affective	−2.957	1.262	−.179	**.020**
Lifestyle	−1.013	1.263	−.074	.348
Antisocial	−.295	1.077	−.023	.752
Constant	211.462	6.270		
Overall regression model	*R* = .20 *R*^*2*^ = .04 *F* (4, 251) = 2.65, *p* = .034			

*Note. N* = 256, significant *p*-values (two-tailed) in bold font.

**Table 5.** Multiple Regression: PCL-R Bivariate and Multipredictor Associations with WAI Bond Score among Indigenous and Non-Indigenous Subgroups.

PCL-R Facet Score Regression model	*Regression and WAI DV*			
	*B*	*SE*	*β*	*p*
DV = WAI bond	Indigenous			
Interpersonal	.720	.817	.100	.380
Affective	−1.243	.816	−.192	.130
Lifestyle	.035	.642	.006	.956
Antisocial	−.621	.611	−.106	.311
Constant	67.799			
Overall regression model	*R* = .209, *R*^*2*^ = .044. *F* (4, 109) = 1.25, *p* = .294			
DV = WAI bond	Non-Indigenous			
Interpersonal	−.284	.701	−.039	.686
Affective	−1.100	.682	−.160	.109
Lifestyle	−.778	.639	−.139	.226
Antisocial	.152	.521	.031	.770
Constant	72.346	3.348		
Overall regression model	*R* = .259, *R*^*2*^ = .067. *F* (4, 140) = 2.45, *p* = .049			

*Note.* Indigenous *n* = 114; Non-Indigenous *n* = 141.

### Incremental Associations of Working Alliance, Psychopathy, and Treatment Change to Recidivism

The final set of analyses examined the associations of working alliance, psychopathy, risk and treatment change to recidivism (*N* = 222). The sample was followed up an average 10.5 years (*SD* = 3.1) in the community post release. The base rate of sexual recidivism was 21.6% (48/222) and violent recidivism, 45.5% (101/222). Table 6 reports bivariate associations between the predictor and recidivism criterion (Harrell’s C), as well as the full predictor model with associations reported (B and e^B^) controlling for all other predictors. Significant, small to moderate C magnitude associations were found for most model predictors in their associations with sexual recidivism, including treatment change (inverse association represented by positive C value); WAI score did not significantly predict sexual recidivism on its own.

**Table 6.** Cox Regression Survival Analysis: Bivariate and Incremental Associations of WAI, Psychopathy, Risk, and Treatment Change to Sexual and Violent Recidivism.

Regression model	*Bivariate*		*Cox regression*					
	*C*	*95% CI*	*B*	*SE*	*Wald*	*p*	*e* ^*B*^	*95% CI*
Model 1	Sexual recidivism							
PCL-R total	.58	.50, .65	.013	.022	0.36	.547	1.013	0.971, 1.057
VRS-SO pretreatment total	.63**	.55, .70	.068	.019	12.51	**<.001**	1.070	1.031, 1.112
VRS-SO change	.61**	.52, .70	−.199	.058	11.59	**<.001**	0.820	0.731, 0.919
WAI total	.52	.44, .60	.010	.005	4.17	**.041**	1.010	1.000, 1.019
Model 2	Violent recidivism							
PCL-R total	.64***	.59, .70	.055	.016	11.95	**< .001**	1.056	1.024, 1.089
VRS-SO pretreatment total	.62***	.56, .67	.045	.014	10.74	**.001**	1.046	1.018, 1.075
VRS-SO change	.57*	.51, .62	−.091	.038	5.89	**.015**	0.913	0.848, 0.983
WAI total	.54	.49, .60	.003	.003	0.87	.351	1.003	0.997, 1.009

*Note. N* = 222 except for Cox regression sexual recidivism model (*N* = 221) owing to treatment cohort entered as a stratum variable. C = Harrel’s C representing bivariate associations between predictor and criterion *p < .05, **p < .01, ***p < .001. Bivariate VRS-SO change association with recidivism conducted employing residualized change score (i.e., controlling for initial pretreatment dynamic score). Cox regression represents incremental associations between each predictor and criterion, controlling for all other predictors in the model; significant *p*-values in bold font. PCL-R: Psychopathy Checklist-Revised; VRS-SO pretreatment total: Violence Risk Scale-Sexual Offense version combined static and pretreatment dynamic total score; VRS-SO change: Violence Risk Scale-Sexual Offense version change score; WAI total: Working Alliance Inventory total score.

Examination of Schoenfeld residuals generated from the Cox regression models demonstrated even patterns of dispersion when plotted against survival time, providing support for the proportional hazards assumption. After treatment cohort was entered as a stratum variable for Cox regression survival analyses, one case was dropped from sexual recidivism analyses (*N* = 221) owing to being censored before the first recidivistic event within a stratum, while all cases could be retained for violent recidivism (*N* = 222). Cohort group comparisons on predictor variables included in the Cox regression models are reported in supplemental materials Table S1; the results of MANOVA with post hoc Tukey beta comparisons demonstrated most differences were small in magnitude (*η*^*2*^ < .06). In the Cox regression model, VRS-SO pretreatment total score (i.e., baseline risk) and treatment change uniquely predicted sexual recidivism, while individual differences in levels of psychopathy did not. Unexpectedly, higher WAI total scores were uniquely significantly associated with increased sexual recidivism. As reported in supplemental analyses, Tables S1–S3, this pattern of multipredictor findings was generally found when separately examining each of the individual WAI subscales, although among the WAI subscales, it was specifically Goal scores that accounted for this unexpected positive association with increased sexual recidivism. For violent recidivism, each model predictor had significant bivariate associations (C) with this outcome, except WAI score, and in the Cox regression model, each variable incremented predictions of this outcome while WAI score did not; again, such associations were observed when examining specific WAI subscales (Tables S2–S4).^1^

As seen in Table 7, similar patterns of predictive associations for PCL-R, VRS-SO risk and change scores with sexual recidivism were observed for Indigenous and non-Indigenous subgroups, limited power notwithstanding; however, only in the non-Indigenous subgroup did WAI score approach a meaningful association in the prediction of sexual recidivism. In supplemental analyses examining WAI subscales (Tables S5–S7), again it was the Goal component of the WAI that had significant positive associations with increased recidivism, and only for non-Indigenous men. For violent recidivism, psychopathy, risk, and change associations demonstrated significant unique associations with this outcome among Indigenous men, while these were smaller in magnitude and less frequently significant for non-Indigenous men. WAI total score was not incrementally predictive of violence for either subgroup, nor were specific WAI subscales in supplemental analyses (Tables S5–S7).

**Table 7.** Cox Regression Survival Analysis: Incremental Associations of WAI, Psychopathy, Risk, and Treatment Change to Sexual and Violent Recidivism among Indigenous and Non-Indigenous Subgroups.

Regression model	*Cox regression*					
	*B*	*SE*	*Wald*	*p*	*e* ^*B*^	*95% CI*
Sexual recidivism	Indigenous					
Model 1a						
PCL-R total	.007	.035	0.39	.844	1.007	0.940, 1.078
VRS-SO pretreatment total	.071	.029	5.77	**.016**	1.073	1.013, 1.137
VRS-SO change	−.306	.099	9.64	**.002**	0.736	0.607, 0.893
WAI total	.010	.008	1.61	.204	1.010	0.995, 1.025
Model 1b	Non-Indigenous					
PCL-R total	.029	.030	0.93	.334	1.029	0.971, 1.091
VRS-SO pretreatment total	.061	.026	5.31	**.021**	1.063	1.009, 1.119
VRS-SO change	−.126	.072	3.07	.080	0.881	0.765, 1.015
WAI total	.013	.007	3.65	.056	1.013	1.000, 1.027
Violent recidivism	Indigenous					
Model 2a						
PCL-R total	.079	.026	9.64	**.002**	1.082	1.030, 1.138
VRS-SO pretreatment total	.060	.020	9.28	**.002**	1.062	1.022, 1.103
VRS-SO change	−.127	.057	4.99	**.026**	0.881	0.788, 0.985
WAI total	.001	.005	0.02	.889	1.001	0.992, 1.010
Model 2b	Non-Indigenous					
PCL-R total	.050	.023	4.94	**.026**	1.052	1.006, 1.099
VRS-SO pretreatment total	.035	.021	2.84	.092	1.035	0.994, 1.078
VRS-SO change	−.052	.053	0.97	.325	0.949	0.855, 1.053
WAI total	.005	.005	1.40	.237	1.005	0.996, 1.015

*Note.* Indigenous *n* = 99; Non-Indigenous *n* = 122 except for Cox regression sexual recidivism model (*n* = 121) owing to treatment cohort entered as a stratum variable. Model and predictor results generated after entering treatment cohort as a stratum variable. Cox regression represents incremental associations between each predictor and criterion, controlling for all other predictors in the model; significant *p*-values in bold font. PCL-R: Psychopathy Checklist-Revised; VRS-SO pretreatment total: Violence Risk Scale-Sexual Offense version combined static and pretreatment dynamic total score; VRS-SO change: Violence Risk Scale-Sexual Offense version change score; WAI total: Working Alliance Inventory total score.

## Discussion

Psychopathy has been long associated with therapeutic pessimism, although recent reviews suggest room for cautious optimism and that evidence informed programs adhering to “what works” may have some potential to benefit high psychopathy men. The present study examined the self-reported working alliance of men attending a high intensity SOTP, and its associations with psychopathy, sexual violence risk, treatment change, and recidivism. The results have implications for the therapeutic engagement of clientele with substantial psychopathic traits in correctional programs and as outlined in Wong’s (2015) 2-component (2-C) RNR-based model for the treatment of psychopathy.

### Working Alliance Associations with Responsivity Variables

Several variables had significant small to moderate associations with WAI indexes that have responsivity implications. Higher levels of verbal and nonverbal cognitive ability could Bode for clearer communication, ability to encode and retain treatment material, understanding of interventions (including homework), and reduced potential for frustration or other roadblocks in individual sessions; hence, its slightly higher associations with Task and Goal, compared to the Bond. Weaker associations with Bond would imply that cognitive ability seemed to have little bearing on the emotional connection between client and therapist. The positive association between employment history and each of the WAI indices may translate into good therapeutic work ethic (per DeSorcy et al., 2020) and possibly gaining more from sessions. The lack of association between most binary diagnostic categories and WAI indices would suggest that diagnoses, particularly those quite prevalent in a correctional sample (e.g., ASPD, SUD), had little bearing on the quality of connection between client and therapist. An exception appeared to be psychosis, which had a very low base rate in the sample, but nonetheless, may reflect that certain psychotic symptoms (e.g., paranoia, suspiciousness, delusions of persecution) can undermine the sense trust and mutual connection that would seem to be essential in an alliance.

### Working Alliance, Sexual Violence Risk, and Treatment Change

Baseline risk on dynamic risk factors was generally not meaningfully associated with WAI scores overall or on each of the subscales. This suggests that even high risk men were capable of developing strong working alliances overall and on each of the task, bond, and goal domains; being high risk did not preclude forming strong working alliances and being meaningfully engaged in therapy. An exception was Treatment Responsivity factor scores association with weaker alliances—in short, this domain assesses attitudes and cognitions supportive of sexual violence, one’s level of insight and understanding, acceptance of responsibility for sexual offending, and attitude toward treatment. It stands to reason that greater problems in this domain could translate into treatment resistance, difficulties in agreement on therapeutic tasks, weaker emotional bonds, and fewer shared goals of treatment.

Changes on the Treatment Responsivity domain were only weakly associated with most indices of the working alliance, while changes in the domains of Sexual Deviance and Criminality had small, significant associations with most WAI indices. What may account for this? Sexual deviance and general antisociality or criminality are the primary domains of risk and need that predict future sexual violence (Hanson & Morton-Bourgon, 2005) and which are problem areas targeted in SOTPs (McGrath et al., 2010). Therapeutic foci such as paraphilic interests, problematic sexual arousal, and other aspects of sexual functioning are personal and sensitive in nature, and require discretion, sensitivity, tact, and openness to promote engagement and therapeutic work in this area; hence, the necessity of a positive working alliance. Moreover, research has demonstrated that the relevance of the sexual deviance construct may be moderated by victim profile (i.e., having greater relevance for men who have sexually offended against children), particularly for Indigenous men on the VRS-SO (Olver et al., 2020), which may account for the differential associations observed between change in this domain and working alliance. Further, it is possible that the bond may have had less relevance than tasks and goals per se when it came to making risk relevant changes for the Indigenous men per DeSorcy et al. (2016).

It is important to keep in mind that these associations were small in magnitude and a very small amount of the variance in risk change (i.e., <5%) can be accounted for by the alliance and its elements alone as operationalized by WAI score; particularly considering this was assessed during the initial months of treatment. Much of SOTP is also group based and there is much individual therapist variability in skill, style, and approach (Marshall et al., 2011). As such, other therapeutic factors that are both general (e.g., group process dynamics) and specific (e.g., group module focus, specific interventions, ancillary programming) would account for remaining variability in these changes. This would also include the ebb and flow of quality of the alliance that could be expected to occur over treatment, particularly an eight–9-month program. Further, a strong alliance towards one’s primary therapist could translate into positive engagement and attitude toward other service providers and the program in general.

### Working Alliance and Psychopathy: Never the Twain Shall Meet?

Consistent with prior research (DeSorcy et al., 2020) and Wong’s (2015) 2-C model, the callous and unemotional features of psychopathy (i.e., PCL-R Affective facet scores), and to some degree, Factor 1 in general (per Daly, 2017), had significant inverse associations with the alliance overall as well as the bond and goal components that were borne out in regression analyses; the relevance of the Affective facet with respect to decreased Bond scores was apparent for both Indigenous and non-Indigenous subgroups. Given that the partialled PCL-R facet associations retained the direction of their association with WAI measures and decreased slightly in magnitude from their respective zero order correlations, it is unlikely that suppressor effects were at work (per Hicks and Patrick (2006)) or that partialling adversely affected interpretation of findings (per Lynam et al., 2006).

The practice implications per C-1 are to manage the Factor 1 characteristics to retain the client in treatment to increase the potential for benefit. Rather than trying to fundamentally alter the character structure of a psychopathic client, several provisions can be made including staff training, documentation of staff-client interactions, maintenance of clear boundaries, clear communication among staff, staff support and supervision, management of countertransference reactions, and the use of motivational and engagement strategies focusing on the task and goal components of the alliance (Wong, 2015; Wong et al., 2012). There are also several practice implications per C-2, specifically: i) use of a dynamic risk measure (e.g., VRS, VRS-SO) to inform treatment intensity (risk principle) and to identify criminogenic targets for intervention (need principle), to monitor treatment progress and to evaluate change; and ii) group and individual services to prioritize dynamic risk factors for correctional populations in general to promote the development of prosocial cognitive, interpersonal, and behavioral skills for risk reduction and promotion of prosocial functioning.

### Psychopathy, Alliance, and Treatment Outcome

With all this in mind it is important to underscore that there were small differences overall between high and low psychopathy men in their aggregate WAI scores; some men, even with substantial psychopathic traits, reported strong working alliances comparable to their less psychopathic peers. Treatment outcome analyses demonstrated that baseline sexual violence risk and treatment change predicted sexual recidivism over time controlling for psychopathy and working alliance, overall (per C-2 of Wong, 2015, model), and across Indigenous and non-Indigenous subgroups; such associations were larger in magnitude and more frequently significant for the Indigenous subgroup. That is, treatment-related changes in risk on areas of criminogenic need were associated with decreased sexual and violent recidivism, and often carried greater weight in regression models. Unexpectedly, higher pretreatment WAI scores (particularly the Goal component) were uniquely significantly associated with sexual recidivism, but only after controlling for baseline risk and change; this association was found with and without controls for psychopathy and it was present only for the non-Indigenous subgroup.

It is important to consider the timing of the WAI administrations and their magnitude of associations with risk change. Working alliance is a responsivity indicator, and on its own, does not appear to have a great deal of risk relevance. Having a positive alliance, especially when measured in the early stages of treatment (i.e., first 3 months in the current sample), can Bode well for future treatment engagement and hence, risk reduction; however, when variance accounted for by actual change and baseline risk measured by the VRS-SO is partialled out of WAI scores, there is remaining risk variance contained within WAI scores not accounted for by these other sources. It is possible that this residual risk variance in WAI scores may reflect exaggerated and unrealistic self-appraisals of treatment engagement when commitment and actual progress is comparatively thin; that is, the individual is talking the talk, but he is not walking the walk. On the surface, strong WAIs are generally positive but there is much variability in the meaning of those scores, especially when measured in a sexual offending population and obtained via self-report, and particularly when the focus of the treatment program is the reduction and prevention of future sexual violence. In other words, there may be a subset of patients who are “faking it” vis-à-vis the alliance or who simply have low insight and high denial about their relationship with their primary therapist; a phenomenon that may have been more likely for the non-Indigenous, and predominantly White, subgroup of men. Given that non-Indigenous men have been found to score higher on the Interpersonal facet than Indigenous men (Olver et al., 2018a), including the current sample, it is possible that such characteristics may have mutually inflated perceptions of the alliance for the non-Indigenous men. Importantly, the present findings also suggest that clinician rated measures can provide a reliable indicator of treatment progress and risk reduction.

### Service Delivery Implications with Indigenous Persons

Although these targeted analyses demonstrated some continuity of WAI correlates with psychopathy and treatment change for Indigenous and non-Indigenous men, the associations still tended to be smaller in magnitude and less consistently significant in the Indigenous subgroup. What the results indicate is that, for Indigenous men, there is a smaller direct association between their self-reported working alliance with their primary therapist, and the amount of change they display in treatment as well as the level of psychopathy traits they exhibit. Our sense is that there are several other relationships with therapeutic relevance that these men have in the institution and within the SOTP, and unique to Indigenous men being that of Elders and cultural programming staff, in addition to the other program personnel for all patients (e.g., treatment module leaders, unit psychologist, parole officer) that could influence treatment buy-in and gain, as noted previously. If nothing else, the relationship with the primary therapist is important, but some of these other relationships may carry differentially greater influence for Indigenous men not captured by WAI ratings. Second, the smaller observed associations between PCL-R facet scores and the working alliance for Indigenous treatment participants (with Affective facet-Bond associations still being most central) may reflect a common thread of a slightly weaker alliance, on average, between Indigenous men and their typically non-Indigenous primary therapist, irrespective of psychopathy. Although keeping in mind, that the WAI ratings on a whole were quite high, this possibility is strengthened by the fact that the highest WAI ratings were observed for non-Indigenous men who were also low psychopathy.

These considerations, notwithstanding, it is important to reiterate the value of general therapeutic factors such as empathy, warmth, genuineness, and positive regard, which intersect with core correctional practices and general responsivity; all clients need this as a platform for service delivery, not all treatment-related personnel will be adept at giving it. That Indigenous and non-Indigenous men made comparable degrees of risk change as evaluated independently on a dynamic risk tool, and that such changes were linked to decreased sexual and violent recidivism independent of the working alliance, imply some common therapeutically relevant risk reduction mechanisms at work (e.g., skills acquisition and usage, risk factor management, release planning, bolstering community supports). It is likely, however, that the relative weight of some of the relational pathways to attaining these common outcomes are different in some capacities.

### Limitations, Conclusions, and Future Directions

There are noteworthy strengths and limitations of the present study with implications for research and practice. First, there were several new or unexamined areas of inquiry targeted by the present study, including: (i) the examination of treatment-related risk change with working alliance, and specifically, in a sexual offending sample, (ii) the association of working alliance with correctional outcomes such as recidivism (controlling for covariates such as risk and psychopathy), (iii) the examination of other responsivity considerations (e.g., cognitive ability, diagnosis) relevant to the working alliance, (iv) nuanced examination of the components of the psychopathy construct as they relate to the alliance, (v) extending research on how the working alliance may relate to client baseline risk, and (vi) conducting parallel strategically selected analyses for Indigenous and non-Indigenous men. Second, the WAI and other self-reports were all completed independent of the VRS-SO and PCL-R; thus, raters did not have knowledge of WAI scores to potentially influence ratings. That the WAIs were also self-report would serve to rule out possibilities that associations with clinician rated measures could be inflated owing to shared method variance. The VRS-SOs and PCL-Rs were also completed independently of each other by trained raters, who were also blind to outcome. Third, the study had comprehensive outcome data from the country’s national reporting service (CPIC) and good duration of follow-up, which coupled with the sizable N, contributed sufficient power to conduct key analyses. Fourth, the study was a prospective examination of the WAI, with the inventory administered in the course of routine clinical practice with the sample followed up post release in real time.

That said, one key limitation was that the WAI was administered at only one time point and during the first 3 months of treatment. WAIs completed later in the course of services have generated stronger associations with outcomes of various types (Flückiger et al., 2018) and this, in part, may have attenuated associations with recidivism. Moreover, the assessment of the alliance at only one time point did not permit examination of changes in the alliance over the course of treatment, or how such changes would link to changes in risk or other program targets, features of psychopathy, or post program outcomes. Aside from Polaschek and Ross (2010) who found positive changes in the alliance to be associated with risk reduction in a sample of violent offenders, to our knowledge, no other research has done this with a treated correctional sample, and none with a treated sexual offending sample. Second, only a single rater was used to examine the working alliance—patient self-report—which has its own set of strengths and limitations (e.g., potential for demand characteristics). The study would have been strengthened further through other rater perspectives, such as therapists, partners and family members, or third-party clinician observer ratings. Importantly, the limited research on alliance within treated sexual offending samples has shown close correspondence between client and therapist ratings, even within different levels of psychopathy (e.g., Walton et al., 2018), which provides some support for the veracity of the WAI ratings in the present study. Third, and relatedly, the present study did not conduct interviews, which could have yielded a greater depth and richness of clinical detail to inform PCL-R ratings, client and therapist perceptions of the alliance, and treatment change.

Finally, given that “the relationship between a therapist and a client is embedded in cultural norms and expectations about the psychotherapist/helper role” (Flückiger et al., 2018, p. 332), further research should examine diversity considerations (e.g., gender, culture, and ethnicity) in the alliance for the client and/or therapist and what bearing this may have on therapeutic outcomes (e.g., DeSorcy et al., 2016). The present study offered an important look at Indigenous heritage as a possible moderator of key effects, demonstrating both important areas of continuity and possible differences. Indigenous heritage is highly diverse, however, including over 600 First Nations bands, the Métis, and Inuit, and the present study did not have the sample size or power to thoroughly interrogate substantive research questions at this level of analysis. The present study also did not have information on the gender or ethnocultural background of the service provider, and thus, could not examine to what extent mutuality in client and therapist characteristics related to the alliance. In conclusion, high psychopathy men with a history of sexual offending have the potential to establish meaningful working alliances and make risk relevant changes if retained in RNR based services. Future research should extend and replicate these findings on high risk-high need treated samples, to inform therapeutic processes and outcomes, to reduce violent victimization and promote public safety and client wellbeing.

## Supplemental Material

Supplemental Material - The Risk, Need, and Responsivity Relevance of Working Alliance in a Sexual Offense Treatment Program: Its Intersection With Psychopathy, Diversity, and Treatment Change Supplemental Material for The Risk, Need, and Responsivity Relevance of Working Alliance in a Sexual Offense Treatment Program: Its Intersection With Psychopathy, Diversity, and Treatment Change by Mark E. Olver, Keira C. Stockdale, and Emily K. Riemer in Sexual Abuse

## Note

1. Controlling for time from index sentence to program admission or time from program discharge to community release did not substantively alter predictor criterion variable associations in Cox regression models.
