# Supplementary material for: The Risk, Need, and Responsivity Relevance of Working Alliance in a Sexual Offense Treatment Program: Its Intersection With Psychopathy, Diversity, and Treatment Change
Source: Sex Abuse. 2023 Apr 24;36(4):383–417. doi: 10.1177/10790632231172161 (PMC11010564; doi:10.1177/10790632231172161)
Supplement: Supplemental Material - The Risk, Need, and Responsivity Relevance of Working Alliance in a Sexual Offense Treatment Program: Its Intersection With Psychopathy, Diversity, and Treatment Change [file sj-pdf-1-sax-10.1177_10790632231172161.pdf]

Supplemental Tables

Table S1

MANOVA with Tukey Beta Post hoc comparisons for SOTP Cohorts on Key Predictor Variables (N = 222)

Measure	1994-1997 cohort	1998-2000 cohort	2001-2003 cohort		
	M [SD] n = 60	M [SD] n = 123	M [SD] n = 39	*F*	η^2
WAI					
Task	68.3 [13.2]	68.0 [10.9]	66.9 [13.2]	0.18	.00
Bond	61.5 [14.7]	61.7 [13.2]	61.5 [16.0]	0.00	.00
Goal	63.4 [12.5]	65.7 [11.9]	66.1 [12.9]	0.85	.01
Total	193.2 [37.1]	195.4 [32.5]	194.5 [40.2]	0.08	.00
PCL-R					
Interpersonal	2.6 [1.9]	2.6 [1.9]	3.2 [2.1]	1.60	.01
Affective	4.5 [2.3] [a]	3.9 [1.9] [a]	5.4 [2.0]	8.28***	.07
Lifestyle	5.5 [3.0]	5.1 [2.3]	6.2 [2.1]	2.67	.02
Antisocial	5.4 [3.3] [a]	4.9 [2.4] [a]	6.5 [2.6]	4.74*	.04
Factor 1	7.1 [3.7] [a]	6.5 [3.3] [a]	8.6 [3.5]	5.44**	.05
Factor 2	10.8 [5.6]	10.1 [4.1] [a]	12.6 [4.2]	4.82**	.04
Total	20.3 [8.4]	17.8 [6.0] [a]	22.8 [6.5]	8.62***	.07
VRS-SO					
Dynamic (pre)	25.5 [7.9] [a,b]	30.9 [5.4]	31.7 [7.0]	16.61***	.13
Dynamic (post)	22.0 [7.5] [a,b]	26.6 [6.0]	27.6 [7.3]	11.71***	.10
Change	3.5 [2.3]	4.3 [3.1]	4.1 [2.5]	1.61	.02
Total (pre)	35.2 [11.3] [a,b]	42.1 [7.6]	44.6 [7.7]	17.00***	.13
Total (post)	31.7 [10.9] [a,b]	37.7 [7.8]	40.5 [8.0]	14.18***	.12
Sexual deviance (pre)	6.8 [4.1] [b]	9.1 [3.8]	8.2 [4.3]	6.73***	.06
Sexual deviance (post)	6.0 [3.6] [b]	7.8 [3.3]	7.1 [3.8]	5.73***	.05
Sexual deviance (change)	0.81 [0.83]	1.2 [1.2]	1.0 [1.0]	3.06*	.03
Criminality (pre)	9.3 [4.2] [b]	10.6 [3.5]	11.5 [2.9]	5.33**	.05
Criminality (post)	8.0 [3.7]	9.3 [3.3]	10.1 [2.8]	5.01**	.04
Criminality (change)	1.2 [1.0] [b]	1.4 [1.2]	1.5 [1.1]	0.89	.01
Txt responsivity (pre)	6.6 [2.4] [b]	7.1 [2.3]	7.9 [2.5]	3.33*	.03
Txt responsivity (post)	5.5 [2.4]	5.8 [2.4]	6.7 [2.4]	3.33*	.03
Txt responsivity (change)	1.2 [0.72] [b]	1.3 [1.2]	1.1 [0.85]	1.01	.01

Note: ***p < .001, p < .01, p < .05, WAI: Working Alliance Inventory; PCL-R: Psychopathy Checklist-Revised; VRS-SO: Violence Risk Scale-Sexual Offense version. [a] = significantly different from the 2001-2003 cohort; [b] = significantly different from the 1998-2000 cohort. η^2 = .01 small, .06 medium, .14 large

Table S2

Cox Regression Survival Analysis: Incremental Associations of WAI Task, Psychopathy, Risk, and Treatment Change to Sexual and Violent Recidivism

Regression model	B	SE	Wald	*p*	Exp(B)	95.0% CI for Exp(B)	
						Lower	Upper
Sexual recidivism							
PCL-R total	.008	.022	.143	.706	1.008	.966	1.053
VRS-SO pretreatment total	.068	.019	12.303	**<.001**	1.070	1.030	1.112
VRS-SO change	-.188	.057	10.819	**.001**	.828	.741	.927
WAI TASK	.019	.013	2.153	.142	1.020	.993	1.047
Violent recidivism							
PCL-R total	.053	.016	11.243	**<.001**	1.055	1.022	1.088
VRS-SO pretreatment total	.044	.014	10.203	**.001**	1.045	1.017	1.074
VRS-SO change	-.086	.037	5.384	**.020**	.917	.853	.987
WAI TASK	.003	.008	.090	.764	1.003	.986	1.019

Note: N = 222 except for Cox regression sexual recidivism model (N = 221) owing to treatment cohort entered as a stratum variable. * $p < .05$, ** $p < .01$, *** $p < .001$. Bivariate VRS-SO change association with recidivism conducted employing residualized change score (i.e., controlling for initial pretreatment dynamic score). Cox regression represents incremental associations between each predictor and criterion, controlling for all other predictors in the model; significant p-values in bold font. PCL-R: Psychopathy Checklist-Revised; VRS-SO pretreatment total: Violence Risk Scale-Sexual Offense version combined static and pretreatment dynamic total score; VRS-SO change: Violence Risk Scale-Sexual Offense version change score; WAI TASK: Working Alliance Inventory Task score.

Table S3

Cox Regression Survival Analysis: Incremental Associations of WAI Bond, Psychopathy, Risk, and Treatment Change to Sexual and Violent Recidivism

Regression model	B	SE	Wald	*p*	Exp(B)	95.0% CI for Exp(B) Lower	Upper
Sexual recidivism							
PCL-R total	.014	.022	.394	.530	1.014	.971	1.059
VRS-SO pretreatment total	.066	.019	12.052	**<.001**	1.068	1.029	1.108
VRS-SO change	-.196	.058	11.215	**<.001**	.822	.733	.922
WAI BOND	.021	.011	3.306	.069	1.021	.998	1.044
Violent recidivism							
PCL-R total	.057	.016	12.987	**<.001**	1.059	1.026	1.092
VRS-SO pretreatment total	.046	.014	11.149	**<.001**	1.047	1.019	1.075
VRS-SO change	-.095	.038	6.329	**.012**	.909	.844	.979
WAI BOND	.011	.007	2.304	.129	1.011	.997	1.026

Note: N = 222 except for Cox regression sexual recidivism model (N = 221) owing to treatment cohort entered as a stratum variable. * $p < .05$, ** $p < .01$, *** $p < .001$. Bivariate VRS-SO change association with recidivism conducted employing residualized change score (i.e., controlling for initial pretreatment dynamic score). Cox regression represents incremental associations between each predictor and criterion, controlling for all other predictors in the model; significant p-values in bold font. PCL-R: Psychopathy Checklist-Revised; VRS-SO pretreatment total: Violence Risk Scale-Sexual Offense version combined static and pretreatment dynamic total score; VRS-SO change: Violence Risk Scale-Sexual Offense version change score; WAI BOND: Working Alliance Inventory Bond score.

Table S4

Cox Regression Survival Analysis: Incremental Associations of WAI Goal, Psychopathy, Risk, and Treatment Change to Sexual and Violent Recidivism

Regression model	B	SE	Wald	*p*	Exp(B)	95.0% CI for Exp(B) Lower	Upper
Sexual recidivism							
PCL-R total	.013	.022	.346	.557	1.013	.971	1.057
VRS-SO pretreatment total	.070	.019	12.796	**<.001**	1.072	1.032	1.114
VRS-SO change	-.204	.059	12.005	**<.001**	.815	.726	.915
WAI GOAL	.034	.014	5.651	**.017**	1.035	1.006	1.065
Violent recidivism							
PCL-R total	.054	.016	11.571	**<.001**	1.055	1.023	1.089
VRS-SO pretreatment total	.045	.014	10.472	**.001**	1.046	1.018	1.074
VRS-SO change	-.089	.038	5.652	**.017**	.914	.850	.984
WAI GOAL	.006	.009	.417	.518	1.006	.989	1.023

Note: $N = 222$ except for Cox regression sexual recidivism model ($N = 221$) owing to treatment cohort entered as a stratum variable. * $p < .05$, ** $p < .01$, *** $p < .001$. Bivariate VRS-SO change association with recidivism conducted employing residualized change score (i.e., controlling for initial pretreatment dynamic score). Cox regression represents incremental associations between each predictor and criterion, controlling for all other predictors in the model; significant p-values in bold font. PCL-R: Psychopathy Checklist-Revised; VRS-SO pretreatment total: Violence Risk Scale-Sexual Offense version combined static and pretreatment dynamic total score; VRS-SO change: Violence Risk Scale-Sexual Offense version change score; WAI GOAL: Working Alliance Inventory Goal score.

Table S5

Cox Regression Survival Analysis: Incremental Associations of WAI Task, Psychopathy, Risk, and Treatment Change to Sexual Recidivism

Regression model	B	SE	Wald	*p*	Exp(B)	95.0% CI for Exp(B) Lower	Upper
Sexual recidivism				Indigenous			
PCL-R total	.006	.035	.031	.861	1.006	.939	1.079
VRS-SO pretreatment total	.070	.030	5.501	**.019**	1.073	1.012	1.137
VRS-SO change	-.285	.094	9.114	**.003**	.752	.625	.905
WAI TASK	.014	.020	.459	.498	1.014	.974	1.055
				Non-Indigenous			
PCL-R total	.025	.029	.697	.404	1.025	.967	1.086
VRS-SO pretreatment total	.062	.026	5.514	**.019**	1.064	1.010	1.120
VRS-SO change	-.111	.070	2.503	.114	.895	.781	1.027
WAI TASK	.035	.019	3.320	.068	1.036	.997	1.076
Violent recidivism				Indigenous			
PCL-R total	.081	.026	9.788	**.002**	1.084	1.031	1.141
VRS-SO pretreatment total	.058	.020	8.600	**.003**	1.059	1.019	1.101
VRS-SO change	-.120	.056	4.513	**.034**	.887	.795	.991
WAI TASK	-.004	.013	.106	.745	.996	.971	1.021
				Non-Indigenous			
PCL-R total	.047	.023	4.390	**.036**	1.048	1.003	1.096
VRS-SO pretreatment total	.034	.021	2.761	.097	1.035	.994	1.077
VRS-SO change	-.046	.052	.773	.379	.955	.862	1.058
WAI TASK	.010	.013	.558	.455	1.010	.984	1.037

Note: Indigenous *n* = 99; Non-Indigenous *n* = 122 except for Cox regression sexual recidivism model (*n* = 121) owing to treatment cohort entered as a stratum variable. Model and predictor results generated after entering treatment cohort as a stratum variable. Cox regression represents incremental associations between each predictor and criterion, controlling for all other predictors in the model; significant p-values in bold font. PCL-R: Psychopathy Checklist-Revised; VRS-SO pretreatment total: Violence Risk Scale-Sexual Offense version combined static and pretreatment dynamic total score; VRS-SO change: Violence Risk Scale-Sexual Offense version change score; WAI TASK: Working Alliance Inventory Task score.

Table S6

Cox Regression Survival Analysis: Incremental Associations of WAI Bond, Psychopathy, Risk, and Treatment Change to Sexual Recidivism

Regression model	B	SE	Wald	*p*	Exp(B)	95.0% CI for Exp(B) Lower	Upper
Sexual recidivism				Indigenous			
PCL-R total	.009	.035	.061	.805	1.009	.942	1.080
VRS-SO pretreatment total	.068	.029	5.473	**.019**	1.070	1.011	1.133
VRS-SO change	-.290	.094	9.436	**.002**	.748	.622	.900
WAI BOND	.022	.018	1.529	.216	1.022	.987	1.059
				Non-Indigenous			
PCL-R total	.025	.030	.695	.405	1.026	.966	1.089
VRS-SO pretreatment total	.057	.026	4.897	**.027**	1.058	1.006	1.113
VRS-SO change	-.125	.074	2.879	.090	.882	.764	1.020
WAI BOND	.021	.015	1.984	.159	1.022	.992	1.052
Violent recidivism				Indigenous			
PCL-R total	.079	.025	9.778	**.002**	1.082	1.030	1.137
VRS-SO pretreatment total	.064	.020	10.646	**.001**	1.066	1.026	1.108
VRS-SO change	-.140	.058	5.866	**.015**	.870	.777	.974
WAI BOND	.013	.011	1.358	.244	1.013	.991	1.035
				Non-Indigenous			
PCL-R total	.051	.023	4.912	**.027**	1.052	1.006	1.101
VRS-SO pretreatment total	.033	.020	2.656	.103	1.034	.993	1.076
VRS-SO change	-.052	.054	.927	.336	.949	.854	1.055
WAI BOND	.012	.011	1.162	.281	1.012	.990	1.034

Note: Indigenous *n* = 99; Non-Indigenous *n* = 122 except for Cox regression sexual recidivism model (*n* = 121) owing to treatment cohort entered as a stratum variable. Model and predictor results generated after entering treatment cohort as a stratum variable. Cox regression represents incremental associations between each predictor and criterion, controlling for all other predictors in the model; significant p-values in bold font. PCL-R: Psychopathy Checklist-Revised; VRS-SO pretreatment total: Violence Risk Scale-Sexual Offense version combined static and pretreatment dynamic total score; VRS-SO change: Violence Risk Scale-Sexual Offense version change score; WAI BOND: Working Alliance Inventory Bond score.

Table S7

Cox Regression Survival Analysis: Incremental Associations of WAI Goal, Psychopathy, Risk, and Treatment Change to Sexual and Violent Recidivism among Indigenous and Non-Indigenous Subgroups

						95.0% CI for Exp(B)	
Regression model	B	SE	Wald	*p*	Exp(B)	Lower	Upper
Sexual recidivism				Indigenous			
PCL-R total	.009	.035	.065	.799	1.009	.943	1.080
VRS-SO pretreatment total	.072	.029	5.958	**.015**	1.074	1.014	1.138
VRS-SO change	-.331	.105	9.994	**.002**	.718	.584	.882
WAI GOAL	.041	.025	2.664	.103	1.042	.992	1.095
				Non-Indigenous			
PCL-R total	.026	.029	.762	.383	1.026	.969	1.086
VRS-SO pretreatment total	.063	.027	5.531	**.019**	1.065	1.011	1.123
VRS-SO change	-.126	.072	3.063	.080	.881	.765	1.015
WAI GOAL	.042	.020	4.452	**.035**	1.043	1.003	1.085
Violent recidivism				Indigenous			
PCL-R total	.081	.026	9.774	**.002**	1.084	1.031	1.140
VRS-SO pretreatment total	.057	.019	8.701	**.003**	1.059	1.019	1.099
VRS-SO change	-.116	.055	4.400	**.036**	.891	.800	.992
WAI GOAL	-.009	.013	.516	.473	.991	.965	1.016
				Non-Indigenous			
PCL-R total	.049	.022	4.872	**.027**	1.051	1.006	1.097
VRS-SO pretreatment total	.036	.021	2.962	.085	1.036	.995	1.079
VRS-SO change	-.054	.053	1.051	.305	.947	.853	1.051
WAI GOAL	.019	.013	2.049	.152	1.019	.993	1.046

Note: Indigenous n = 99; Non-Indigenous n = 122 except for Cox regression sexual recidivism model (n = 121) owing to treatment cohort entered as a stratum variable. Model and predictor results generated after entering treatment cohort as a stratum variable. Cox regression represents incremental associations between each predictor and criterion, controlling for all other predictors in the model; significant p-values in bold font. PCL-R: Psychopathy Checklist-Revised; VRS-SO pretreatment total: Violence Risk Scale-Sexual Offense version combined static and pretreatment dynamic total score; VRS-SO change: Violence Risk Scale-Sexual Offense version change score; WAI GOAL: Working Alliance Inventory Goal score.
